# Chronic Disease Management in Children Based on the Five Domains of Health

**DOI:** 10.1155/2013/978198

**Published:** 2013-04-09

**Authors:** Wing Lung Alvin So

**Affiliations:** The School of Medicine, The University of Melbourne, Parkville, VIC 3010, Australia

## Abstract

Through a case study of a child with cystic fibrosis, the interactions among various domains of health have been discussed—namely, biomedical, physical, psychological/behavioural, and social. In pediatrics, development is another key domain relevant to the management of a chronic disease. An individualised management plan for this case has been outlined, and consideration of this framework may be worthwhile when managing other paediatric patients with chronic disease. Patient empowerment and parental education, as well as good co-ordination of health service delivery, are imperative to holistic patient care.

## 1. Introduction

Similar to other areas of medicine, the psychosocial health of a paediatric patient interacts with their organic disease. This paper illustrates how the presence of a genetic disease, which can be understood under a chronic disease framework, has an influence on thoughts and emotions of the patient. The resultant behaviour then affects the progress of theirorganic disease. An understanding of the perpetuation of this dynamic process and its impact on the patient's development is the key to effective management.

## 2. Case Study

SW was a seven-year-old boy with cystic fibrosis (CF). His organic disease was complicated by oppositional defiant disorder, social withdrawal, and a difficult family environment. 

SW was suspected to have CF following immunoreactive trypsin test in routine neonatal screening. This was confirmed by DNA analysis and sweat test at 6 weeks of age, and the results showed that SW was homozygous for Δ508 mutation. Recurrent chest infections, often complicated by empyema, resulted in frequent hospitalisation of long duration. Furthermore, he frequently required “tune-up” antibiotics therapy in a tertiary paediatric hospital in Melbourne. Despite optimal treatment with chest physiotherapy and prophylactic antibiotics, SW had severe wheeze and lethargy. Recently, he developed features consistent with bronchiectasis in chest X-ray. Socially, he was not active at home and school. 

In addition, SW suffered from pancreatic insufficiency. He had frequent abdominal pain and steatorrhoea, although the family tried to maintain a low-fat diet. SW often refused to take his pancreatic enzyme supplements as he disliked the taste, and his mother reported the use of “physical force” to make SW comply with his medication. Furthermore, he actively refused other requests by his mother, such as tidying his bedroom. In contrary, SW's grandmother was permissive towards his demands. SW often lost his temper at home and blamed his grandmother for his own mistakes. SW was referred to a child psychiatrist, and a diagnosis of oppositional defiant disorder was made.

The suboptimal control of severe steatorrhoea often resulted in soiling. On one occasion, SW soiled at a classmate's party, and he had since been under the shadow of a psychological trauma. He began to employ a “hold-on” strategy, which eventually led to functional constipation and encopresis. SW was teased in school, often with name calling. He often cried at home following fights at school. Peer relationships and academic performance were poor at school.

Due to poor management of his chronic disease, SW had ongoing growth problems (less than 3rd percentile for height and weight). The poor social relationships in school originated from the birthday party incident further complicated SW's inactivity in the school playground. In addition, his mother reported that SW was reluctant to play with children of his age in family and friend gatherings, although his global development had always been normal. 

SW lived with his mother and grandmother in suburban Melbourne. After school hours, SW spent most of the time at home with his grandmother, as his mother returned to full-time work as a taxi driver when SW was 12 months old. SW's parents separated, and his father rarely visited him. SW's mother found both work and home very stressful and claimed that “more cigarettes helped.” Outside home, she worked long-hour shifts; at home she constantly needed to find ways to make SW comply with his medication, often involving physical punishment. However, when his mother was not home, SW knew that he could use his tantrum strategy on his permissive grandmother to avoid taking medication.

## 3. Discussion

The complex interaction between biological and psychosocial development of a paediatric patient with a genetic disease can be understood under the framework of the International Classification of Functioning, Disability and Health (ICF). Under this framework, the impact of a chronic disease can be divided into biomedical, physical, psychological, and social components. Biomedically, SW suffers from respiratory and gastrointestinal symptoms due to CF. Physically, SW has low activity levels as a result of his respiratory impairments. Psychologically, SW has low self-esteem and oppositional traits from CF and the associated poor medication compliance. Similarly, social participation in school is restricted due to recurrent hospitalisation and poor relationships with classmates. Family environment is inadequate to support his needs. Development should be an additional feature for consideration in pediatrics.

### 3.1. Medical Issues Related to Psychosocial Issues

Poor growth from a chronic disease contributes to poor self-esteem and bullying at school [1]. In addition, CF itself has a close association with psychiatric illness such as depression [2–5]. In a child, this often presents with behavioural disturbance, irritability, and anhedonia, rather than the “typical” somatic symptoms. Therefore, it is essential to monitor for and treat depressive symptoms, which may be masked by the oppositional defiant disorder. Long-term medical adherence is crucial in slowing the progress of functional deterioration of CF, though this may be a stressor for SW. He may see this as a “routine” imposed by his mother, or he may see this as being different from his peers. 

### 3.2. Psychological Issues Related to Medical Issues

In the case of SW, the psychological disturbance following the party soiling episode may contribute to “hold-on” functional constipation and encopresis. The poor social network at school and poor psychological well-being are contributing factors for SW's inactivity, further limiting his functional capacity and reducing the level of biological health. The experience of distress may be somatised to become a panic-attack-like episode. This may be misdiagnosed as a respiratory exacerbation of CF. Moreover, SW's noncompliance to medication may have a psychological origin, as he may use this as an attempt to “take control” over his illness or that he is in denial. Social withdrawal, poor academic performance, and a sense of helplessness from CF further exacerbate this maladaptive defense mechanism. However, SW is probably mature enough to have a basic understanding of his illness and how treatment can help. Forming therapeutic alliance with SW and his carers is important to empower SW to develop more mature coping mechanisms.

### 3.3. Role of Parents and Family

Parents have a major impact in SW's development. Firstly, SW's mother smoking in the house can deteriorate SW's respiratory symptoms. Besides, the practice of physical punishment by the authoritarian mother to improve medication compliance induces fear and hostility in SW, who may respond more aggressively to this stimulus [6]. In addition, the mother serves as an aggressive model for SW and may potentiate violent acts [7]. On the other hand, SW's grandmother is too permissive, as medication adherence is crucial in controlling SW's pancreatic insufficiency, which has medical as well as psychosocial implications [8, 9]. Furthermore, SW may feel neglected by this style of parenting, and the feeling of insecurity may result in more undesirable behaviour, such as blaming his grandmother for his own mistakes, to seek attention. This may exacerbate his oppositional disorder. The ideal attitude to optimise medication compliance may involve the immediate application of negative reinforcement following tantrum, as well as positive reinforcement following appropriate behaviour.

### 3.4. Behavioural Development

Multiple factors such as being brought up in a single-parent family, sub-optimal parenting, oppositional behavior, and lack of social support at school diminish the self-worth of SW and potentially increase his likelihood of undertaking risky behaviour during adolescence, for example, smoking, the use of recreational drugs, and unsafe sexual practice [10–12]. This will have a profound effect on his future medical and psychosocial health. Moreover, he may progress to the more severe conduct disorder, which may persist in adulthood as antisocial behavior [13, 14]. If SW fails to develop a sense of self-worth by refining skills in social interaction and taking control of his disease, he may continue to employ maladaptive defense mechanisms throughout his life [15]. Ongoing advances in the medical management of CF mean that SW is likely to have a longer life expectancy; thus the long-term care should be multidisciplinary addressing his medical, psychiatric, and education needs [16, 17].

## 4. Management Plan

**Figure 1:**
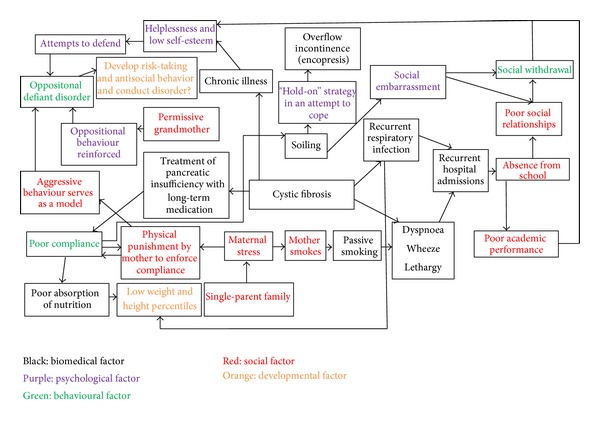
The multifactorial interactions of the disease of SW.

Based on the study of complex interactions among the biomedical, psychological, behavioural, social, and developmental factors of SW's illness, which has been summarised in Figure 1, his management plan may include the following issues.

 Biomedical
  (i) Respiratory System
    (a) Continue daily physiotherapy and prophylactic antibiotics to prevent bacterial conlonisation.
    (b) Regular exercise. Encourage SW to undertake sport in school, as it can also improve his relationship with his classmates.
  (ii) Pancreatic Insufficiency
    (a) Improve medication adherence by education of the mother and grandmother, through the application of classical learning theory. When SW shows aggressive behaviour, immediately tell him to stop it and give brief reasons. If he continues, have a “time-out” session in which he is left alone. Reapply if necessary for negative reinforcement. If SW has done the right thing, give him a reward such as a sticker or take him out to the park. Empower SW in the process: explain to him in plain language his disease and what he can do to make him feel better.
    (b) Parenting groups may also help by sharing different experience.
    (c) Maintain a balanced diet (low fat, high fibre), liaise with dietitian.
  (iii) Encopresis
    (a) Regular toileting with appropriate reward. When necessary, laxatives such as polyethylene glycol preparations may be useful.
    (b) Referral to the continence clinic if not controlled by the above measures.
  (iv) Low Weight and Height
     Overall good medical control of CF.
 Psychological
  (i) Oppositional Defiant Disorder
    (a) Parent management therapy (PMT) and family therapy to alter the parenting styles of mother and grandmother to discourage oppositional behaviour and encourage appropriate behavior.
    (b) Followup with child psychiatrist to monitor for depressive symptoms and adjust treatment accordingly.
    (c) Social worker and the school counseling service may also be beneficial [18].
 Social
  (i) Family
    (a) Coping strategies for SW's mother: single-mother support groups, effective communication to the grandmother, involve neighbours and other relatives in the care of SW.
    (b) Liaise with social work and respite care may be useful.
    (c) Advice regarding her smoking as it is harmful to her health and can worsen SW's pulmonary condition—motivational interview has a role.
  (ii) School
    (a) Provide special classes if SW cannot keep up with his academic study due to hospitalisation.
    (b) Engage school teachers for school activities and improve peer relationships, consolidate friendship bonding by organising overnight stay in classmates' houses.
  (iii) Coordination of Management
     The multidisciplinary management of SW should be orchestrated by his GP and further refined by his paediatrician.

## 5. Conclusion

The multidirectional interactions between the disease and illness of SW lay the foundation to formulate a holistic management plan, addressing the medical, physical, psychosocial, and developmental needs of SW. Likewise, it is essential to educate and empower the patient and his family during the process. Since growing is a dynamic process, the management should be adjusted at intervals to better suit the needs of SW. This multidisciplinary multidimensional consideration may form a useful framework for chronic disease management in pediatrics.
